# Different Dose Regimens of Intravenous Tranexamic Acid in Adolescent Spinal Deformity Surgery: A Systematic Review and Meta-Analysis

**DOI:** 10.1155/2020/3101358

**Published:** 2020-11-28

**Authors:** Zhencheng Xiong, Kexin Wu, Jiayu Zhang, Delong Leng, Ziyi Yu, Chi Zhang, Ping Yi

**Affiliations:** ^1^Institute of Medical Technology, Peking University Health Science Center, Beijing, China; ^2^Peking University Third Hospital, Beijing, China; ^3^Postgraduate School, Dalian Medical University, Dalian, China; ^4^Graduate School of Peking Union Medical College, Chinese Academy of Medical Sciences, Beijing, China; ^5^Department of Clinical Medicine, School of Clinical Medicine, Bengbu Medical College, Bengbu, China; ^6^Department of Urology, Peking University Cancer Hospital & Institute, Beijing, China; ^7^Department of Orthopedics, Peking University International Hospital, Beijing, China; ^8^School of Chinese Materia Medica, Beijing University of Chinese Medicine, Beijing, China; ^9^Department of Spine Surgery, China-Japan Friendship Hospital, Beijing, China

## Abstract

**Objective:**

To evaluate the efficacy and safety of different dose regimens of intravenous (IV) tranexamic acid (TXA) in adolescent spinal deformity surgery.

**Methods:**

Two researchers independently searched multiple databases, including PubMed, Embase, Cochrane Library, and Web of Science to find studies that met the inclusion criteria. A meta-analysis was performed based on the guidelines of the *Cochrane Reviewer's Handbook*.

**Results:**

Six randomized controlled trials (RCTs) and eleven non-RCTs were identified, including 1148 patients. According to different dose regimens of IV TXA, the included studies were divided into the high-dose group and the low-dose group. Compared with placebo, both groups had less total blood loss (TBL) (high dose: WMD = −1737.55, 95% CI: (-2247.16, -1227.94), *P* < 0.001, *I*^2^ = 0%; low dose: WMD = −528.67, 95% CI: (-666.06, -391.28), *P* < 0.001, *I*^2^ = 0%), intraoperative blood loss (IBL) (high dose: WMD = −301.48, 95% CI: (-524.3, -78.66), *P* = 0.008, *I*^2^ = 60.3%; low dose: WMD = −751.14, 95% CI: (-967.21, -535.08), *P* < 0.001, *I*^2^ = 0%), and blood transfusion rates (high dose: RR = 0.19, 95% CI: (0.1, 0.37), *P* < 0.001, *I*^2^ = 0%; low dose: RR = 0.4, 95% CI: (0.18, 0.91), *P* = 0.029, *I*^2^ = 57%). High-dose IV TXA use was associated with more vertebral fusion segments (WMD = 0.53, 95% CI: (0.23, 0.82), *P* < 0.001, *I*^2^ = 31.2%). Low-dose IV TXA use was associated with shorter operative time (WMD = −18.43, 95% CI: (-26.68, -10.17), *P* < 0.001, *I*^2^ = 0%).

**Conclusion:**

High-dose and low-dose IV TXA were effective in reducing TBL, IBL, and blood transfusion rates without increasing complications in adolescent patients undergoing spinal deformity surgery. Low-dose IV TXA was effective in reducing the operative time. Both the high-dose and low-dose groups had similar preoperative and postoperative Hb levels compared to the control group.

## 1. Introduction

Perioperative blood loss is a major problem in surgery, especially in complex high-risk surgical procedures, such as spinal deformity surgery [1]. As we all know, the posterior column and three column osteotomies may be the more commonly used procedures in spinal deformity surgery, although it may bring significant risks [2]. Some spinal diseases require spinal deformity surgery for further deterioration, including degenerative scoliosis, adolescent idiopathic scoliosis (AIS), degenerative lumbar kyphosis, posttraumatic kyphosis, and Duchenne muscular dystrophy (DMD) [3]. AIS, a complex three-dimensional deformity of the spine, is defined as a lateral curvature of the spine > 10° in the coronal plane [4]. AIS correction surgery and other spinal deformity surgeries are associated with significant blood loss. Increased blood loss brings many clinical risks, including hemodynamic instability, progressive multiple organ dysfunction, transfusion reaction, hypersensitivity, and increased risk for infection [5]. Therefore, how to reduce perioperative blood loss during spinal deformity surgery has become a hot topic for spinal surgeons. At present, many hemostatic drugs, including tranexamic acid (TXA), are used to prevent significant blood loss in spinal deformity surgery [2].

TXA, a synthetic lysine analogue, exerts an antifibrinolytic effect through binding to the lysine-binding sites on plasminogen molecules and inhibiting fibrinolysis [6]. Some clinical studies and meta-analyses show that intravenous (IV) TXA can reduce blood loss and allogeneic blood transfusion without the high risk of complications such as pulmonary embolism (PE), deep vein thrombosis (DVT), or other [1–5, 7–23]. For IV TXA in spinal deformity surgery, high-dose and low-dose stratification can be performed. In one study, Raman et al. [24] demonstrated that high-dose TXA was more effective than low-dose TXA in reducing blood loss and blood transfusion requirements in spinal deformity surgery. The high-dose group in this study used three dosage regimens [24]. However, the number of studies that directly compare high-dose TXA and low-dose TXA is limited. The optimal dosage scheme of TXA in spinal deformity surgery is still controversial. Therefore, we conducted this meta-analysis to evaluate the efficacy and safety of different dose regimens of IV TXA in adolescent spinal deformity surgery. This meta-analysis sets the definition of high-dose IV TXA to include any dose ≥ 20 mg/kg or >1 g. On the contrary, it is a low-dose regimen. We divided the studies that met the criteria into a high-dose group and a low-dose group and conducted a subgroup analysis of the dose for the same outcome measurements.

## 2. Materials and Methods

### 2.1. Search Strategy

To obtain all relevant studies, two researchers independently searched multiple databases according to Cochrane Collaboration guidelines, such as PubMed (1966 to April 1, 2020), Embase (1980 to April 1, 2020), Cochrane Library (1980 to April 1, 2020), and Web of Science (1965 to April 1, 2020). Literature was searched with the MeSH terms and corresponding keywords (connecting via Boolean operators “AND or OR”), including “tranexamic acid or TXA”, “intravenous”, “spine deformity”, “spine surgery”, “scoliosis”, “spinal deformity surgery”, and “adolescent”. We set the search language limit to English. Two researchers reviewed potential articles based on the titles and abstracts and identified the full text of eligible articles according to the inclusion and exclusion criteria. Then, by reading the full text, we further filter the selected literature. Besides, the reference lists of all retrieved studies were screened to identify potentially relevant studies. If there was disagreeable literature between the two researchers, our research team would discuss to reach a consensus. This meta-analysis was conducted based on the Preferred Reporting Items for Systematic Reviews and Meta-Analyses (PRISMA) statement [25].

### 2.2. Study Selection

Inclusion criteria for this meta-analysis are the following: (1) all studies involved the comparison of the effect of IV TXA versus a placebo or control group in patients undergoing adolescent spinal deformity surgery; (2) randomized controlled trials (RCTs) or non-RCTs meet the criteria; (3) the study population has a diagnosis of spinal deformities, such as AIS, posttraumatic kyphosis, and degenerative lumbar scoliosis; (4) the study population all had spinal instrumentation and fusion surgery in professional medical institutions due to spinal deformities; (5) the study population had no history of spinal surgery and no history of a bleeding disorder or antifibrinolytic therapy; and (6) data on relevant outcome measurements can be extracted.

The following were excluded from this meta-analysis: (1) studies were not suitable with the inclusion criteria; (2) the types of studies were case reports, case series, conference abstracts, reviews, letters, and editorials; (3) the patient's age is classified as a child, adult, middle adult, or elderly; and (4) data of studies cannot be extracted.

### 2.3. Data Extraction

Data was extracted independently by two researchers, and then, another researcher collected the data using a spreadsheet. Disagreements in the data extraction process were resolved after discussion. The following general characteristics were extracted: first author, publication year, country, study type, number of participants (IV TXA: control), surgical procedure, age, body mass index (BMI), gender, intervention (IV TXA: control), outcome measurements, and transfusion criteria.

### 2.4. Quality Assessment

According to the Cochrane Handbook for Systematic Reviews, two researchers independently assessed the quality of each included RCT [26]. A “risk of bias” table was created with the following elements: (i) random sequence generation; (ii) allocation concealment; (iii) blinding of participant and personnel; (iv) blinding of outcome assessment; (v) incomplete outcome data; (vi) selective reporting; and (vii) other bias. Each of the above sections has a higher risk of bias, a lower risk of bias, and an unclear risk of bias, depending on the actual content of each RCT [26].

The Newcastle-Ottawa scale (NOS) was used to assess the quality of included non-RCTs [27]. In this scale, there are three major items (selection, comparability, and outcome), which can be subdivided into eight detailed quality items. In “selection” and “outcome,” each quality item can be awarded a maximum of one star. In “comparability,” up to two stars can be given. One star represents one point, and the higher the score, the higher the quality assessment [27]. We set low-quality, moderate-quality, and high-quality studies at a score of 0-3, 4-6, and 7-9, respectively.

### 2.5. Statistical Analysis

Data for the same outcome measurements in all studies were summarized in the same table. Outcome measurements were divided into subgroups according to the dosage regimen or recording time. The continuous data was analyzed by using weighted mean difference (WMD) and 95% confidence interval (CI), such as total blood loss (TBL), intraoperative blood loss (IBL), preoperative and postoperative hemoglobin (Hb) levels, operative time, number of vertebral segments fused, and estimated blood loss per fusion segment. Dichotomous data, such as blood transfusion rates, was analyzed using the risk ratio (RR) and 95% CI. The heterogeneity of the included studies was evaluated using the *χ*^2^ test and *I*^2^ test. When the value of *I*^2^ is 25%, 50%, and 75%, it is regarded as low, medium, and high heterogeneity [28]. When *I*^2^ > 50%, *P* < 0.1, we performed a random-effects model; otherwise, a fixed-effects model was performed [28]. All statistical analyses were undertaken using STATA software version 16.0 (Stata Corporation, College Station, Texas, USA) and RevMan 5.3 for Windows (Cochrane Collaboration, Oxford, UK). If *P* < 0.05, the results of this meta-analysis were considered statistically significant.

## 3. Results

### 3.1. Search Results

**Figure 1:**
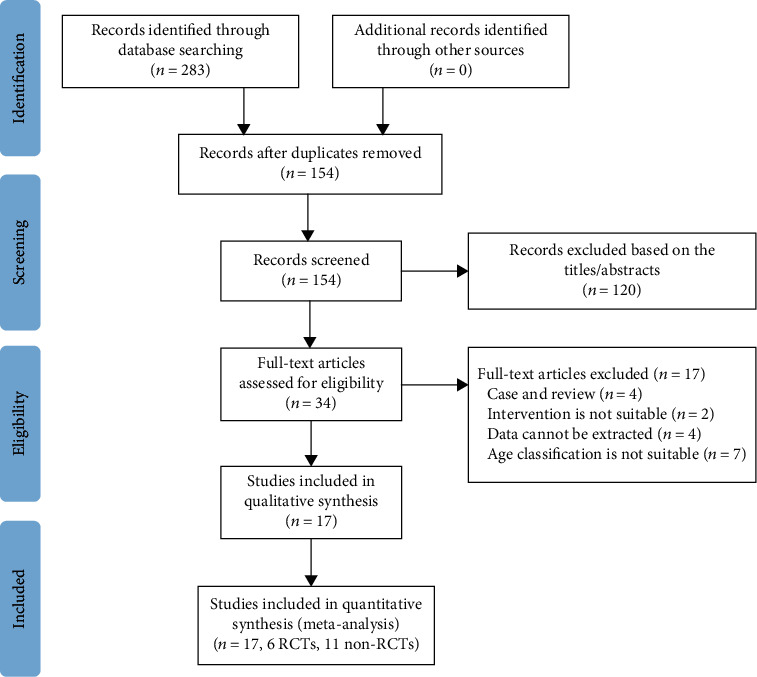
Flow diagram of the study selection process for the meta-analysis.

A total of 283 potentially relevant articles were generated, including PubMed (*n* = 48), Cochrane Library (*n* = 38), Web of Science (*n* = 43), and Embase (*n* = 154) based on search strategy and inclusion criteria. After screening the titles and abstracts, 120 articles were excluded due to duplicate articles, conference abstracts, case reports, letters, reviews, and irrelevant studies. Based on the inclusion and exclusion criteria, the full text of the remaining 34 articles was evaluated for eligibility. Finally, 6 RCTs and 11 non-RCTs were included in this meta-analysis [4, 5, 7–21].  Figure 1 is a flow diagram of the study selection.

### 3.2. Study Characteristics

Six RCTs and 11 non-RCTs involving 1148 patients were analyzed in this meta-analysis [4, 5, 7–21]. All included studies were published between 2001 and 2019 [4, 5, 7–21]. The characteristics of all included studies are shown in Table 1. The efficacy and safety of different dose regimens of IV TXA in adolescent spinal deformity surgery had been compared in all studies. A total of 9 studies were high-dose regimens [4, 5, 8–10, 15–17, 19], and the remaining 8 studies were low-dose regimens [7, 11–14, 18, 20, 21]. Of these 9 studies, 3 studies were RCTs [5, 8, 10], and the rest were non-RCTs [4, 9, 15–17, 19]. Among these studies with the high-dose regimen, there are 6 studies with the same dose regimen, all with a loading dose of 100 mg/kg infused before skin incision and a maintenance dose of 10 mg/kg/h [8, 9, 15–17, 19]. Of these 8 studies, 3 studies were RCTs [7, 13, 21], and the rest were non-RCTs [11, 12, 14, 18, 20]. Among them, there are 4 studies with the same dose regimen, all with a loading dose of 10 mg/kg infused before skin incision and a maintenance dose of 1 mg/kg/h [7, 13, 18, 21]. Among the 1148 adolescent patients, the mean ages ranged from 13.5 to 21.6, and the sample sizes of the IV TXA group ranged from 18 to 71 [4, 5, 7–21]. Of the 17 studies, the main diagnosis of 14 studies was AIS [4, 5, 7, 8, 10–14, 17–21], one study was DMD [9], and the remaining were other spinal deformities [15, 16]. The surgical method used in 15 studies [4, 5, 7, 9, 11–21] was posterior spinal fusion. These studies mainly include three surgical methods, all of which were spinal deformity surgery [4, 5, 7–21].

### 3.3. Risk of Bias

**Figure 2:**
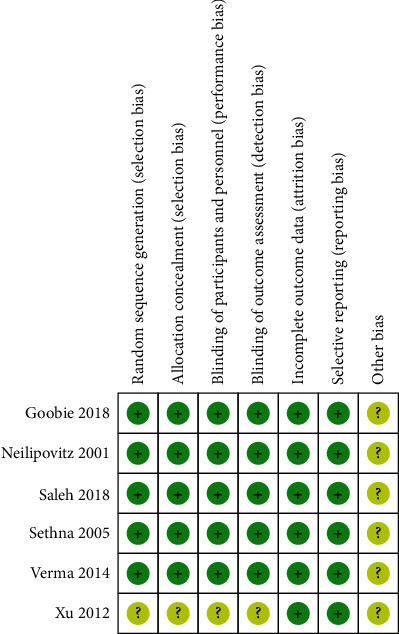
Risk of bias summary: +: low risk of bias; −: high risk of bias; ?: bias unclear.

Figure 2 shows the risk of bias assessment for the 6 RCTs [5, 7, 8, 10, 13, 21]. A total of 5 RCTs were considered to have a low risk of bias [5, 7, 8, 13, 21]. Random sequence generation, allocation concealment, blinding of participants and personnel, and blinding of outcome assessment were found in five studies [5, 7, 8, 13, 21]. None of the six RCTs found selective reports [5, 7, 8, 10, 13, 21].

Table 2 shows the risk of bias assessment for the 11 non-RCTs [4, 9, 11, 12, 14–20]. According to the NOS, 5 studies received 8 points [11, 14, 16, 17, 20], and 6 studies received 7 points [4, 9, 12, 15, 18, 19], indicating that the quality of included studies was acceptable.

### 3.4. Results of the Meta-analysis

#### 3.4.1. TBL

**Figure 3:**
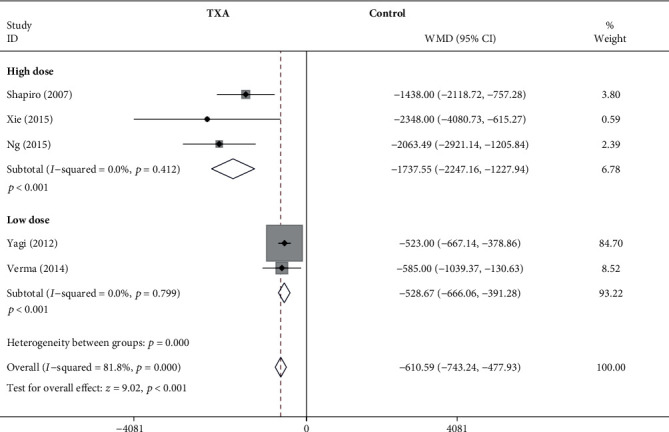
Forest plot showing the effect of high-dose IV TXA regimen compared with low-dose IV TXA regimen on TBL during adolescent spinal deformity surgery. IV: intravenous; TXA: tranexamic acid; TBL: total blood loss; WMD: weighted mean difference.

As shown in Figure 3, the forest plot shows the effect of the high-dose IV TXA regimen compared with the low-dose IV TXA regimen on TBL during adolescent spinal deformity surgery. Five studies provided TBL as the primary outcome measurement [9, 11, 13, 16, 17]. TBL was divided into 2 subgroups according to different dosage regimens. A total of 3 studies (205 patients) [9, 16, 17] provided data on TBL for the high-dose regimen, and 2 studies (189 patients) [11, 13] provided data on TBL for the low-dose regimen. Because there was no significant heterogeneity (*I*^2^ < 50%), a fixed-effects model was used. There was a statistically significant difference in TBL between the high-dose TXA group and the control group based on the results of the pooled analysis (WMD = −1737.55, 95% CI: (-2247.16, -1227.94), *P* < 0.001, *I*^2^ = 0%). And there was a statistically significant difference in TBL between the low-dose TXA group and the control group (WMD = −528.67, 95% CI: (-666.06, -391.28), *P* < 0.001, *I*^2^ = 0%).

#### 3.4.2. IBL

**Figure 4:**
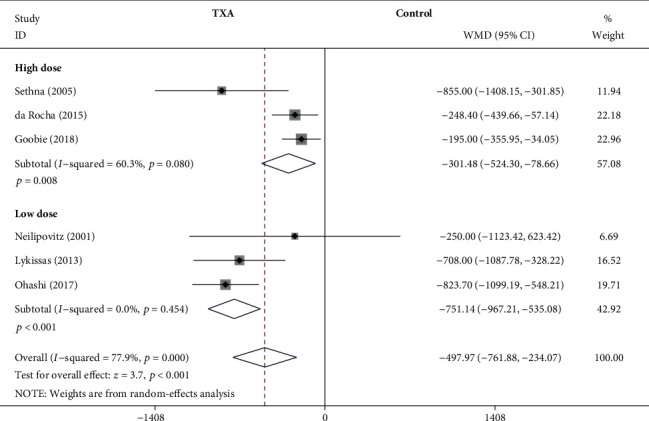
Forest plot showing the effect of high-dose IV TXA regimen compared with low-dose IV TXA regimen on IBL during adolescent spinal deformity surgery. IBL: intraoperative blood loss.

As shown in Figure 4, the forest plot shows the effect of the high-dose IV TXA regimen compared with the low-dose IV TXA regimen on IBL during adolescent spinal deformity surgery. Six studies provided IBL as the primary outcome measurement [5, 7, 8, 12, 15, 20]. IBL was divided into 2 subgroups according to different dosage regimens. A total of 3 studies (195 patients) [5, 8, 15] provided data on IBL for the high-dose regimen, and 3 studies (152 patients) [7, 12, 20] provided data on IBL for the low-dose regimen. Because of the significant heterogeneity (*I*^2^ > 50%, *P* < 0.1), a random-effects model was used. There was a statistically significant difference in IBL between the high-dose TXA group and the control group based on the results of the pooled analysis (WMD = −301.48, 95% CI: (-524.3, -78.66), *P* = 0.008, *I*^2^ = 60.3%). And there was a statistically significant difference in IBL between the low-dose TXA group and the control group (WMD = −751.14, 95% CI: (-967.21, -535.08), *P* < 0.001, *I*^2^ = 0%).

#### 3.4.3. Operative Time

**Figure 5:**
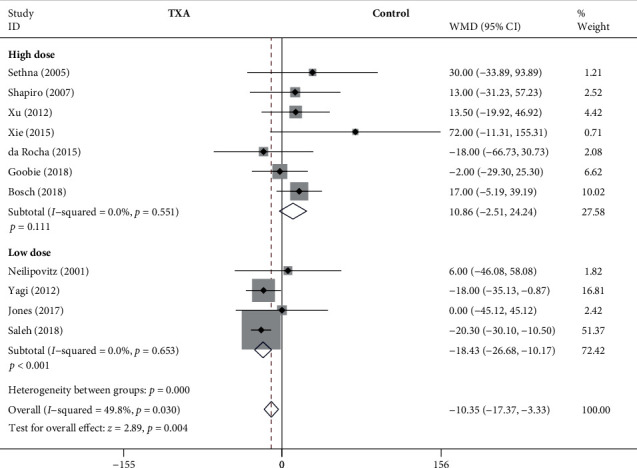
Forest plot showing the effect of high-dose IV TXA regimen compared with low-dose IV TXA regimen on operative time during adolescent spinal deformity surgery.

As shown in Figure 5, the forest plot shows the effect of the high-dose IV TXA regimen compared with the low-dose IV TXA regimen on operative time during adolescent spinal deformity surgery. Eleven studies provided operative time as the secondary outcome measurement [4, 5, 7–11, 15, 16, 18, 21]. Operative time was divided into 2 subgroups according to different dosage regimens. A total of 7 studies (438 patients) [4, 5, 8–10, 15, 16] provided data on operative time for the high-dose regimen, and 4 studies (232 patients) [7, 11, 18, 21] provided data on operative time for the low-dose regimen. Because there was no significant heterogeneity (*I*^2^ < 50%), a fixed-effects model was used. There were no statistically significant differences in operative time between the high-dose TXA group and the control group based on the results of the pooled analysis (WMD = 10.86, 95% CI: (-2.51, 24.24), *P* = 0.111, *I*^2^ = 0%). However, there was a statistically significant difference in operative time between the low-dose TXA group and the control group (WMD = −18.43, 95% CI: (-26.68, -10.17), *P* < 0.001, *I*^2^ = 0%).

#### 3.4.4. Blood Transfusion Rate

**Figure 6:**
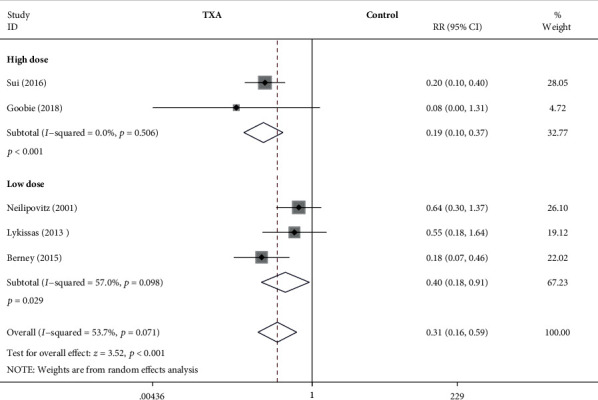
Forest plot showing the effect of high-dose IV TXA regimen compared with low-dose IV TXA regimen on blood transfusion rate during adolescent spinal deformity surgery. RR: risk ratio.

As shown in Figure 6, the forest plot shows the effect of the high-dose IV TXA regimen compared with the low-dose IV TXA regimen on the blood transfusion rate during adolescent spinal deformity surgery. Five studies provided the blood transfusion rate as the secondary outcome measurement [5, 7, 12, 14, 19]. The blood transfusion rate was divided into 2 subgroups according to different dosage regimens. A total of 2 studies (248 patients) [5, 19] provided data on the blood transfusion rate for the high-dose regimen, and 3 studies (145 patients) [7, 12, 14] provided data on the blood transfusion rate for the low-dose regimen. Because of the significant heterogeneity (*I*^2^ > 50%, *P* < 0.1), a random-effects model was used. There was a statistically significant difference in the blood transfusion rate between the high-dose TXA group and the control group (RR = 0.19, 95% CI: (0.1, 0.37), *P* < 0.001, *I*^2^ = 0%). And there was a statistically significant difference in the blood transfusion rate between the low-dose TXA group and the control group based on the results of the pooled analysis (RR = 0.4, 95% CI: (0.18, 0.91), *P* = 0.029, *I*^2^ = 57%).

#### 3.4.5. Preoperative and Postoperative Hb Levels

**Figure 7:**
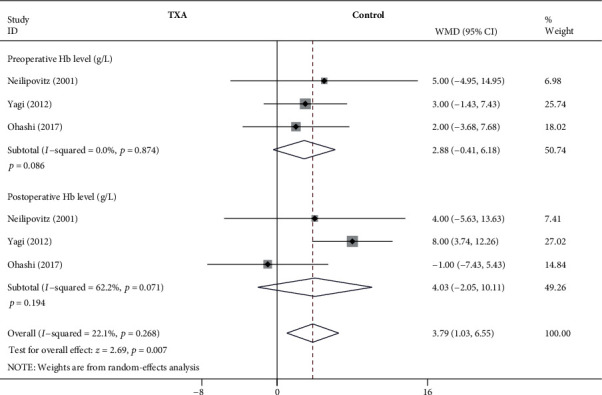
Forest plot showing the effect of low-dose IV TXA on Hb level compared with the control group during adolescent spinal deformity surgery. Hb: hemoglobin.

As shown in Figure 7, the forest plot shows the effect of low-dose IV TXA on Hb level compared with the control group during adolescent spinal deformity surgery. Three studies provided preoperative and postoperative Hb levels as the secondary outcome measurement [7, 11, 20]. Hb was divided into 2 subgroups according to different time points. A total of 3 studies (209 patients) [7, 11, 20] provided data on the preoperative Hb level and postoperative 24 h Hb level. Because of the significant heterogeneity (*I*^2^ > 50%, *P* < 0.1), a random-effects model was used. There were no statistically significant differences in preoperative and postoperative Hb levels between the low-dose TXA group and the control group based on the results of the pooled analysis (preoperative: WMD = 2.88, 95% CI: (-0.41, 6.18), *P* = 0.086, *I*^2^ = 0%; postoperative 24 h: WMD = 4.03, 95% CI: (-2.05, 10.11), *P* = 0.194, *I*^2^ = 62.2%).

#### 3.4.6. Number of Vertebral Segments Fused

**Figure 8:**
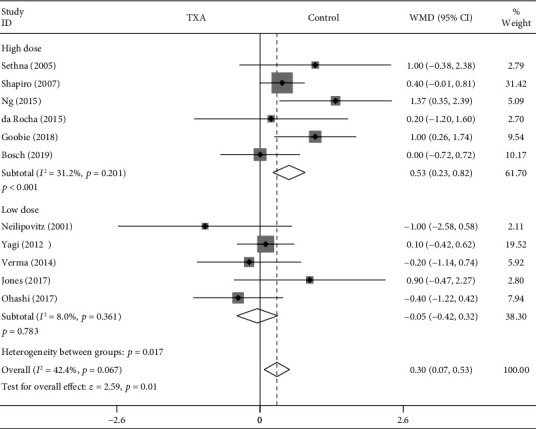
Forest plot showing the effect of high-dose IV TXA regimen compared with low-dose IV TXA regimen on the number of vertebral segments fused during adolescent spinal deformity surgery.

As shown in Figure 8, the forest plot shows the effect of the high-dose IV TXA regimen compared with the low-dose IV TXA regimen on the number of vertebral segments fused during adolescent spinal deformity surgery. Eleven studies provided the number of vertebral segments fused as the secondary outcome measurement [4, 5, 7–9, 11, 13, 15, 17, 18, 20]. The number of vertebral segments fused was divided into 2 subgroups according to different dosage regimens. A total of 6 studies (429 patients) [4, 5, 8, 9, 15, 17] provided data on the number of vertebral segments fused for the high-dose regimen, and 5 studies (328 patients) [7, 11, 13, 18, 20] provided data on the number of vertebral segments fused for the low-dose regimen. Because there was no significant heterogeneity (*I*^2^ < 50%), a fixed-effects model was used. There were no statistically significant differences in the number of vertebral segments fused between the low-dose TXA group and the control group based on the results of the pooled analysis (WMD = −0.05, 95% CI: (-0.42, 0.32), *P* = 0.783, *I*^2^ = 8%). However, there was a statistically significant difference in the number of vertebral segments fused between the high-dose TXA group and the control group (WMD = 0.53, 95% CI: (0.23, 0.82), *P* < 0.001, *I*^2^ = 31.2%).

#### 3.4.7. Estimated Blood Loss per Fusion Segment

**Figure 9:**
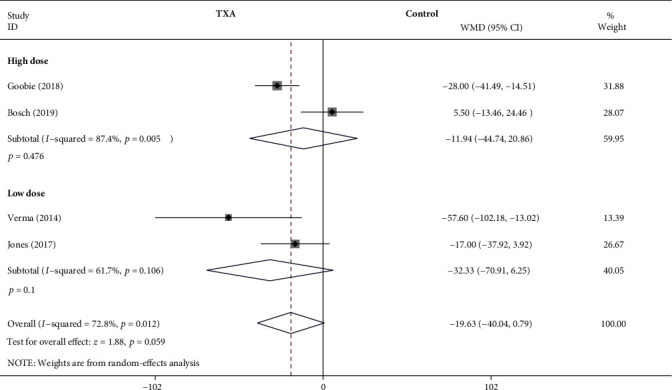
Forest plot showing the effect of high-dose IV TXA regimen compared with low-dose IV TXA regimen on estimated blood loss per fusion level during adolescent spinal deformity surgery.

As shown in Figure 9, the forest plot shows the effect of the high-dose IV TXA regimen compared with the low-dose IV TXA regimen on estimated blood loss per fusion segment during adolescent spinal deformity surgery. Four studies provided estimated blood loss per fusion segment as the secondary outcome measurement [4, 5, 13, 18]. Estimated blood loss per fusion segment was divided into 2 subgroups according to different dosage regimens. A total of 2 studies (199 patients) [4, 5] provided data on estimated blood loss per fusion segment for the high-dose regimen, and 2 studies (108 patients) [13, 18] provided data on estimated blood loss per fusion segment for the low-dose regimen. Because of the significant heterogeneity (*I*^2^ > 50%, *P* < 0.1), a random-effects model was used. There were no statistically significant differences in estimated blood loss per fusion segment between the high-dose TXA group and the control group based on the results of the pooled analysis (WMD = −11.94, 95% CI: (-44.74, 20.86), *P* = 0.476, *I*^2^ = 87.4%). And there were no statistically significant differences in estimated blood loss per fusion segment between the low-dose TXA group and the control group (WMD = −32.33, 95% CI: (-70.91, 6.25), *P* = 0.1, *I*^2^ = 61.7%).

#### 3.4.8. Adverse Event

None of the included 17 studies reported adverse events, such as DVT/PE, allergic reaction, angina, myocardial infarction, new-onset arrhythmia, pneumonia, wound problem, and urinary tract infection. Comparing the differences in adverse events of different dosage regimens, more high-quality research is still needed.

### 3.5. Publication Bias

**Figure 10:**
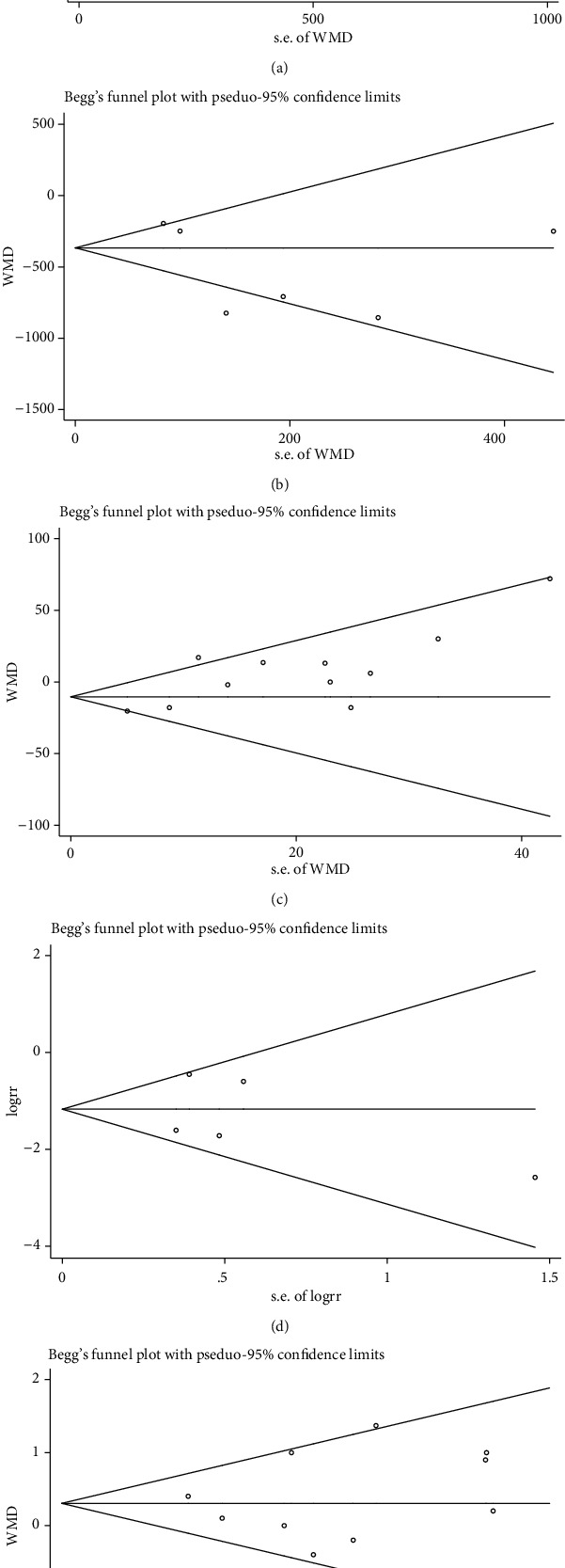
Begg's test for publication bias: (a) TBL, (b) IBL, (c) operative time, (d) blood transfusion rate, and (e) number of vertebral segments fused.

The funnel plot, Begg's funnel plot, and Egger's test were used to assess publication bias and were usually performed in at least 10 studies. No publication bias was detected by Begg's test due to all *P* values > 0.05 for TBL, IBL, operative time, blood transfusion rate, or number of vertebral segments fused (Begg's test, *P* = 0.221, *P* = 0.707, *P* = 0.213, *P* = 0.806, and *P* = 0.876, respectively). The remaining outcome measurements were not suitable for Begg's test and Egger's test due to too few studies.  Figure 10 shows Begg's test for publication bias.

### 3.6. Sensitivity Analysis

**Figure 11:**
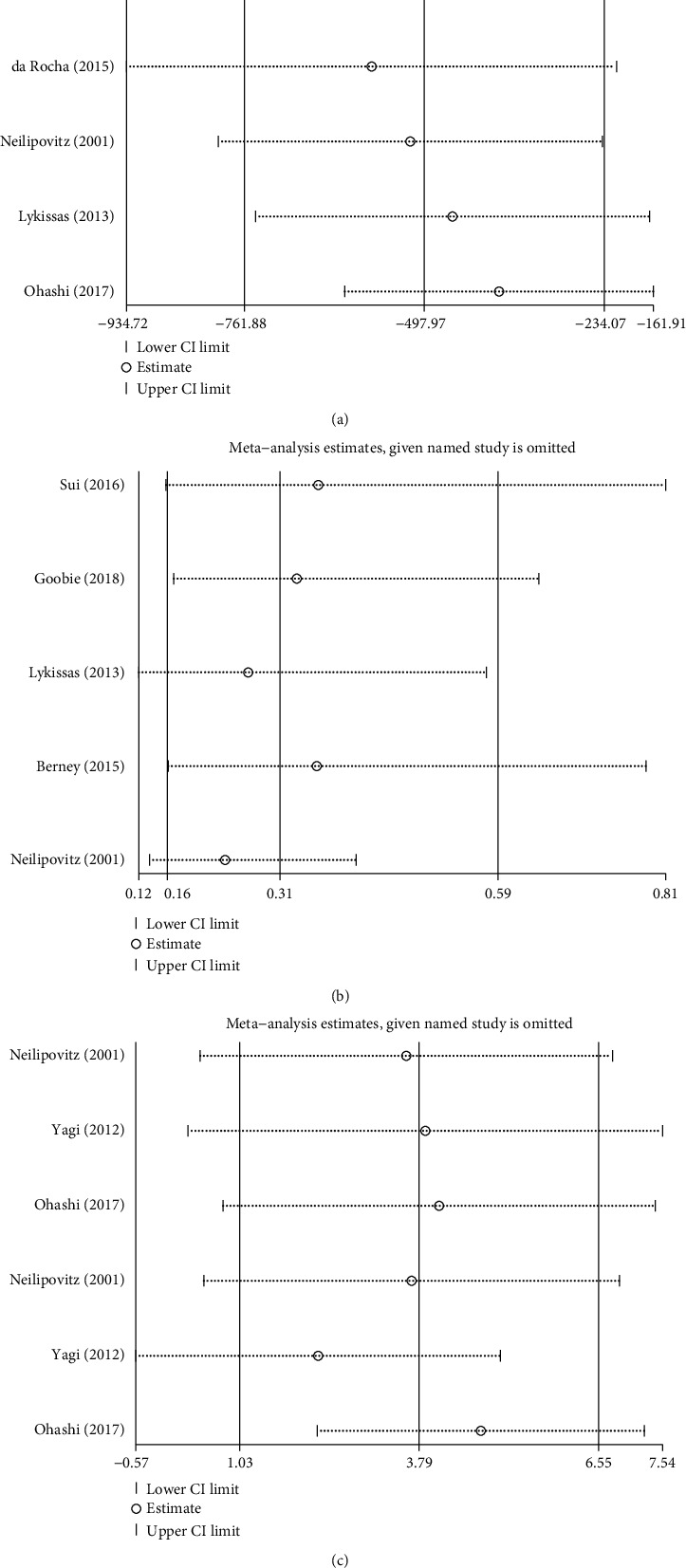
Sensitivity analysis for confirmation of the stability of the pooled result: (a) IBL, (b) blood transfusion rate, and (c) preoperative and postoperative Hb levels.

Sensitivity analysis was conducted to assess the stability of the pooled result. Based on the results of the pooled analysis, high heterogeneity (*I*^2^ > 50%, *P* < 0.1) was found in IBL, blood transfusion rate, preoperative and postoperative Hb levels, and estimated blood loss per fusion segment. Because only two studies have been included in different subgroups of estimated blood loss per fusion segment, its high heterogeneity may be due to the limited number of studies. For other outcome measures, we found that when excluding any study, the results did not find significant changes, thus confirming the robustness and reliability of the results of this meta-analysis (Figure 11). The sources of high heterogeneity in this meta-analysis may be as follows: (1) the number of the studies included in each subgroup is limited; (2) the differences between the included studies are inherent; (3) the doses, methods of use, and operators are not exactly the same among the included studies; (4) the sample size and the collection time of outcome measures are not exactly the same among the included studies; and (5) the diagnosis and operation of the patients are not the same.

## 4. Discussion

This meta-analysis is ultimately a step to popularize the general application of TXA in spinal deformity surgery to reduce adolescent scoliosis. The results of the pooled analysis showed that the significant effect of reducing perioperative blood loss and blood transfusion rate might make TXA the drug of choice. TXA, as an antifibrinolytic drug, is currently mainly used in orthopedic, cardiac, and spine surgery to treat or prevent excessive perioperative blood loss [6]. TXA was administered through a variety of routes, including IV, topical, and oral. IV TXA is usually given intravenously at a certain loading dose before incision and a certain maintenance dose until the skin closure [1–5, 7–23]. Due to the difference in the loading dose and maintenance dose, the clinical effect of TXA may be significantly different. Raman et al. [24] demonstrated that high-dose TXA (loading dose: 30-50 mg/kg, maintenance dose: 1-5 mg/kg/h) was more effective than low-dose TXA (loading dose: 10-20 mg/kg, maintenance dose: 1-2 mg/kg/h) in reducing blood loss and blood transfusion requirement in adult spinal deformity surgery. Grant et al. [29] demonstrated that the use of high-dose TXA (loading dose: 20 mg/kg, maintenance dose: 10 mg/kg/h) resulted in a 50% reduction in transfusion requirements for AIS. Johnson et al. [30] demonstrated that high-dose TXA (loading dose: 50 mg/kg, maintenance dose: 5 mg/kg/h) was more effective than low-dose TXA (loading dose: 10 mg/kg, maintenance dose: 1 mg/kg/h) in reducing blood loss and transfusion requirements in spinal deformity surgery. Based on the above research results, we can see that different dosage regimens of IV TXA may produce significantly different clinical results. Therefore, the optimal dosage regimens of TXA are still a clinical problem to be solved by more high-quality RCTs in the future.

The dosage regimen of TXA should be considered when weighing risks and benefits. At present, the commonly used low-dose regimen is the loading dose of 10 mg/kg and the maintenance dose of 1 mg/kg/h [7, 13, 18, 21]. For example, Neilipovitz et al. [7] demonstrated that the administration of low-dose TXA in patients with AIS undergoing posterior spinal fusion surgery had the potential to reduce perioperative blood transfusion requirements. In a study published in 2018 with the same dosage regimen, the same conclusion was found [31]. These studies reported not only the benefits of low-dose TXA but also the risks. Choi et al. [2] found that there were 2 cases of DVT/PE and 2 cases of allergic reaction in the TXA group, but not in the control group. Peters et al. [1] found that there was one case of PE in the TXA group, but not in the control group. On the contrary, the commonly used high-dose regimen is the loading dose of 100 mg/kg and the maintenance dose of 10 mg/kg/h [8, 9, 15–17, 19]. For example, Sethna et al. [8] demonstrated that intraoperative administration of high-dose TXA significantly reduced blood loss during spinal deformity surgery in AIS. The same result has been confirmed in the research in recent years [16–18]. These studies reported not only the benefits of high-dose TXA but also the risks. Kaabachi et al. [32] found that there were 11 cases of vomiting in the TXA group, but not in the control group. Based on the few studies above, it is speculated that low-dose TXA may have more complications than high-dose TXA. However, such speculative results are not rigorous, so more research is still needed in the future to compare the complications of TXA with different dosage regimens.

The application of TXA has rich experience in orthopedic surgery in our hospital. For example, Yue et al. [33] found that TXA effectively reduced the blood loss of unicompartmental knee arthroplasty in patients with anemia, reducing the rate of blood transfusion, without increasing the risk of DVT. Zhu et al. [34] found that the use of TXA in total hip arthroplasty can reduce the blood transfusion rate and reduce TBL and IBL, without increasing the risk of thrombosis. Our surgery team also adopted a low-dose regimen (loading dose: 10 mg/kg; maintenance dose: 1 mg/kg/h) in spinal deformity surgery. Preliminary trials found that TXA effectively reduced the blood transfusion rate and TBL without increasing the risk of thrombosis. However, the optimal dosage regimens of IV TXA in spinal deformity surgery are still controversial. The relationship between the TXA dose and blood loss control is unclear. Therefore, more high-quality RCTs are still needed in the future to explore the optimal dosage regimens of TXA, not only in adolescent spinal deformity surgery but also for different surgical methods.

### 4.1. Limitations

Because of the quantity and quality of the included studies, this meta-analysis has some limitations. Firstly, the number of included RCTs is quite limited, and many studies have incomplete data and relatively low quality. Secondly, the start and end times of the loading and maintenance doses of TXA in different studies are not the same. Thirdly, the loading dose and maintenance dose of TXA in the same dosage regimen are not completely consistent. Fourthly, surgical methods, disease diagnosis, and transfusion criteria of the same dosage regimen are not completely consistent. Finally, the number of non-RCTs is relatively large, and the heterogeneity of some results is relatively high.

## 5. Conclusion

This meta-analysis compares the effects of TXA with different dosage regimens in adolescent spinal deformity surgery. The results of the above analysis indicated that high-dose and low-dose IV TXA were effective in reducing TBL, IBL, and blood transfusion rates without increasing complications in adolescent patients undergoing spinal deformity surgery. Low-dose IV TXA was effective in reducing the operative time. Both the high-dose and low-dose groups had similar preoperative and postoperative Hb levels compared to the control group. Due to the limited number and quality of studies related to some outcome measurements, more high-quality RCTs are still needed in the future to supplement existing conclusions.

## Figures and Tables

**Table 1:** Characteristics of all the trials included in the meta-analysis.

Study	Country	Study type	Mean age (years) T:C	No. of patients T:C	Male T:C	Mean BMI (kg/m^2^) T:C	Disease diagnosis	Surgical methods	TXA dosing (loading + maintenance)	Transfusion criteria	Outcome measures
Neilipovitz et al., [7]	Canada	RCT	14.1/13.7	22/18	12/5	NP	AIS	PSF	10 mg/kg + 1 mg/kg/h	Hb < 7 g/dL	(2), (3), (4), (5), and (6)
Sethna et al., [8]	USA	RCT	13.6/14.0	23/21	17/13	NP	AIS	Spinal fusion	100 mg/kg + 10 mg/kg/h	NP	(2), (3), and (6)
Xu et al., [10]	China	RCT	19.1/20.4	20/20	12/7	NP	AIS	Spinal fusion	20 mg/kg + 10 mg/kg/h	NP	(3)
Verma et al., [13]	USA	RCT	15.3/15.01	36/47	4/16	21.19/21.92	AIS	PSF	10 mg/kg + 1 mg/kg/h	NP	(1), (6), and (7)
Goobie et al., [5]	USA	RCT	14.9/14.7	56/55	10/13	21.1/22.2	AIS	PSF	50 mg/kg + 10 mg/kg/h	NP	(2), (3), (4), (6), and (7)
Saleh et al., [21]	Egypt	RCT	14.6/14.6	25/25	11/11	NP	AIS	PSF	10 mg/kg + 1 mg/kg/h	NP	(3)
Shapiro et al., [7]	USA	RCS	13.9/14.0	20/36	NP	NP	DMD	PSF	100 mg/kg + 10 mg/kg/h	NP	(1), (3), and (6)
Yagi et al., [11]	Japan	RCS	15.2/15.5	43/63	3/4	NP	AIS	PSF	1 g + 100 mg/h	Hb < 7 g/dL	(1), (3), (5), and (6)
Lykissas et al., [12]	USA	RCS	14.7/13.5	25/24	4/2	NP	AIS	PSF	100 mg + 10 mg/h	Hb < 7 g/dL	(2), (4)
Xie et al., [16]	China	RCS	18.9/18.6	26/33	11/15	NP	Spinal deformity	PSF	100 mg/kg + 10 mg/kg/h	NP	(1), (3)
Berney et al., [14]	Ireland	RCS	15.3/16.4	31/25	9/10	20.3/21.2	AIS	PSF with pedicle screws	15 mg/kg + 10 mg/kg/h	Hb < 7 g/dL	(4)
Ng et al., [17]	China	RCS	15.16/15.31	55/35	NP	19.03/17.76	AIS	PSF with pedicle screws	100 mg/kg + 10 mg/kg/h	Hb < 8 g/dL	(1), (6)
da Rocha et al., [15]	Brazil	RCS	18.0/21.6	21/19	NP	NP	Spinal deformity	PSF with pedicle screws	100 mg/kg + 30 mg/kg/h	NP	(2), (3), and (6)
Sui et al., [18]	China	RCS	15.5/16.2	71/66	22/21	17.1/16.9	AIS	PSF with pedicle screws	100 mg/kg + 10 mg/kg/h	NP	(4)
Jones et al., [19]	USA	RCS	16.1/15.2	18/18	2/3	22.2/20.2	AIS	PSF	10 mg/kg + 1 mg/kg/h	NP	(3), (6), and (7)
Ohashi et al., [20]	Japan	RCS	15.1/14.8	30/33	1/5	NP	AIS	PSF	1 g + 100 mg/h	NP	(2), (5), and (6)
Bosch et al., [4]	USA	RCS	13.7/13.5	30/58	4/11	NP	AIS	PSF	30 mg/kg + 10 mg/kg/h	Hb < 7 g/dL	(3), (6), and (7)

T: TXA group; C: control group. TXA: tranexamic acid; RCT: randomized controlled trial; RCS: retrospective controlled study; BMI: body mass index; AIS: adolescent idiopathic scoliosis; DMD: Duchenne muscular dystrophy; PSF: posterior spinal fusion; Hb: hemoglobin; NP: not provided. Outcome measures: (1) total blood loss; (2) intraoperative blood loss; (3) operative time; (4) blood transfusion rate; (5) Hb level; (6) number of vertebral segments fused; (7) estimated blood loss per fusion segment.

**Table 2:** The Newcastle-Ottawa Scale (NOS) for assessing the quality of non-RCTs.

Study	Selection	Comparability	Outcomes	Total scores (maximum 9)
Shapiro et al., [9]	2	2	3	7
Yagi et al., [11]	4	2	2	8
Lykissas et al., [12]	3	2	2	7
Xie et al., [16]	3	2	3	8
Berney et al., [14]	4	2	2	8
Ng et al., [17]	4	2	2	8
da Rocha et al., [15]	2	2	3	7
Sui et al., [18]	3	2	2	7
Jones et al., [19]	3	2	2	7
Ohashi et al., [20]	4	2	2	8
Bosch et al., [4]	3	2	2	7

## Data Availability

The data supporting this meta-analysis is from previously reported studies and datasets, which have been cited.
